# New Insight into the Crayfish *Procambarus clarkii* (Girard, 1852) (Crustacea, Cambaridae): A Morphometric Combined Approach to Describe the Case of a Mediterranean Population

**DOI:** 10.3390/ani14243558

**Published:** 2024-12-10

**Authors:** Noemi Pascale, Ilenia Azzena, Chiara Locci, Ilaria Deplano, Flavio Orrù, Cesare Puzzi, Francesco Are, Fabio Scarpa, Daria Sanna, Marco Casu

**Affiliations:** 1 Department of Chemical, Physical, Mathematical and Natural Sciences, University of Sassari, Via Vienna 2, 07100 Sassari, SS, Italy; 2 Department of Veterinary Medicine, University of Sassari, Via Vienna 2, 07100 Sassari, SS, Italy; iazzena@uniss.it (I.A.); c.locci3@phd.uniss.it (C.L.); arefran@tiscali.it (F.A.); marcasu@uniss.it (M.C.); 3 Department of Biomedical Sciences, University of Sassari, Viale San Pietro 43/b, 07100 Sassari, SS, Italyfscarpa@uniss.it (F.S.); 4 GRAIA—Gestione Ricerca Ambientale Ittica Acque, Via Repubblica 1, 21020 Varano Borghi, VA, Italy; flaorru@gmail.com (F.O.); info@graia.eu (C.P.)

**Keywords:** *Procambarus clarkii*, invasive alien species (IAS), morphometry, reproductive-linked morphotypes, taxonomic and diagnostic analysis, combined approach

## Abstract

We adopted a morphometric approach to provide statistical support for the description of two different morphotypes (I, reproductive, II, non-reproductive) firstly observed in *Procambarus clarkii* specimens caught in a population from Sardinia Island (western Mediterranean). The morphometric study was preceded by molecular taxonomic identification using the mitochondrial Cytochrome C Oxidase subunit I (COI) gene. The presence or absence of the pathogen *Aphanomyces astaci*, responsible for the plague, was also investigated using the ribosomal Internal Transcribed Spacer (ITS) marker. The estimation of the size at onset of maturity (SOM) was further performed in males. Finally, the population structure was examined. Our results confirmed the entirety of the individuals as *P. clarkii*, and the absence of the pathogen *A. astaci*. Morphological analysis revealed a significant correlation between chela and carapace lengths, while intra- and inter-sexual dimorphism in the chela length confirmed the occurrence of the two morphotypes. The SOM in males was established between 35.0 and 37.1 mm carapace length. Finally, the size-population structure showed a Gaussian distribution. The recognition of the two distinct morphotypes in the crayfish could be useful as an additional tool not only to identify the reproductive period of this invasive crustacean but also to enrich the guidelines for the correct taxonomic identification of the species.

## Simple Summary

In the present work, we adopted morphometry to investigate the presence of two different morphotypes in the highly invasive crustacean *Procambarus clarkii*, the Louisiana crayfish, caught in an isolated Mediterranean population so far never investigated. We distinguished two morphotypes: in the “form I”, or the reproductive form, crayfish exhibit enhanced secondary sexual features, such as gonopods and chelae; in the “form II”, animals are equipped with smaller chelae and poorly calcified gonopods. We further estimated by morphometry the size at onset of maturity (SOM) in males of the Louisiana crayfish, between 35.0 and 37.1 mm of carapace length. Morphometric analysis was preceded by molecular techniques used for a correct taxonomic attribution of the crayfish here analyzed. Furthermore, molecular analysis was also applied to detect the presence of the pathogenic fungus, *Aphanomyces astaci*, in the tissues of crayfish. Our study is useful to enrich the biological knowledge of this highly invasive crustacean, whose spread is severely endangering freshwater ecosystems.

## 1. Introduction

The Mediterranean region is considered as a hotspot for bioinvasions [1], and it is threatened by several non-indigenous species currently expanding their ranges and increasing their abundances [2]. Indeed, among the various elements threatening biodiversity, invasive alien species (IAS) are ranked as the second greatest cause of species endangerment and extinction after habitat destruction [3,4]. Aliens’ impacts can be defined as a measurable change to the properties of an ecosystem [5,6], challenging the conservation of biodiversity and natural resources [7]. In such a context, the genus *Procambarus* Ortmann, 1905 (Crustacea: Cambaridae) encompasses several invasive alien species (IASs) well established in Europe [8,9]. Among them, the Louisiana crayfish or red swamp crayfish *Procambarus clarkii* (Girard, 1852) is an extremely invasive colonizer [10], even listed among the 100 worst invasive alien species in the world [10,11]. This crayfish is native to Mexico and south-central United States of America (USA) [12,13], and it was intentionally introduced to Europe for its food value [10,12] and significance in aquaculture [14]. Additionally, its colorful appearance made it popular as an ornamental species in the pet trade [15]. The invasion of European waterbodies began with its introduction to Spain in 1973 for aquaculture purposes [10], and then the species spread across Europe, becoming abundant in different nations [16]. In Italy, its presence was registered from the 1970s onwards and recorded throughout the peninsula [17,18,19,20,21,22,23,24], including the main Italian islands [13,24,25,26,27,28,29,30]. Furthermore, a new record of the crayfish also in the seawaters of Basilicata, Calabria, and Lazio (central-south Italy) has recently been noted [9]. *Procambarus clarkii* has a huge potential for invasiveness, exhibiting *r*-selected features and rapid growth rates [31,32]. Many studies highlighted its clear impact as a keystone species, due to its feeding activity, which mainly involves vegetation, invertebrates, and vertebrates. Introduced crayfish populations are known to negatively impact several members of freshwater communities, outcompeting native crustaceans [33] and reducing the abundance and distribution of many fish and amphibians [34,35,36,37,38,39], aquatic insects [40], and the biomass and biodiversity of macrophytes [14,41,42]. Moreover, due to its ability to completely transform habitats as an ecosystem engineer (sensu [43]), *P. clarkii* is considered a tertiary to secondary burrower [44,45,46,47]. This behavior allows it to overcome unfavorable conditions in both native and invaded habitats [10,16]. Finally, the crayfish is a natural vector of the oomycete *Aphanomyces astaci*, the causative agent of the so-called crayfish plague, a disease responsible for the decimation and near-extinction of many native crayfish populations in Europe, Asia, and South America [8,48,49,50,51,52]. For example, in Italy, *Austropotamobius pallipes* has recently been classified as endangered (EN) according to the IUCN Red List (last assessment 14 April 2010 https://www.iucnredlist.org/es/species/2430/9438817).

In light of this alarming expansion, an early detection of *P. clarkii* presence and a deep knowledge of its potential for dispersal and reproductive behavior are crucial to minimize its harmful impact. *Procambarus clarkii* adults exhibit a cyclical dimorphism between their reproductive (form I) and non-reproductive (form II) morphotypes [53,54,55,56,57,58,59]. This alternation can be easily seen in form I males, which have larger chelae, hooks on the third and fourth pairs of pereopods, and more calcified, whitish gonopods, as well as in form II males, characterized by smaller chelae, the absence of hooks, and less calcified copulatory pleopods [58,59]. Evidence of this phenomenon was also provided for females [58], but it appeared to be less remarkable compared to males [58,60]. As explained in the literature, the cyclical event interests both genders in the species *P. clarkii*, but in particular males. Once specimens reach maturity, it is possible to observe, through the ecdysis process, the alternation of two different morphotypes within the biological cycle of the crayfish. Form I corresponds to the sexually active crayfish and is maintained throughout the reproductive period; it involves some important morphological changes, particularly evident in males at the level of the claws. In this phase, in fact, they lengthen and become stronger. The general coloring of the body initially appears in pale and dull tones, greyish and sometimes brown, changing to green. In form I, the body takes on a uniform dark red shade, with ruby red spiny tubercles standing out across the entire exoskeleton [60]. In females, however, these changes are less spectacular, and they simply involve an enlargement of the claws, the presence of a cornified annulus ventralis (sperm storage site), and different color patterns, which are typically red in form I and brown, changing to green in form II [59]. Under normal conditions, a few weeks after mating (in females about three after the eggs hatch), the crayfish undergoes a molt, reverting back to form II, sexually inactive: the claws are shorter and thinner, the color is less accentuated, the absence of hooks is noted, and the gonopods of the male are not very sclerified. Form I will reappear, with a molt, during the next reproductive period. These events can occur multiple times over the course of a year [60]. Some authors also described the cyclical color pattern as linked to the morphotypes’ alternation [60,61,62], but no surveys on this topic are available at the moment. Under these circumstances, distinguishing and detecting the presence of these two alternative morphotypes in *P. clarkii* early could represent one of the most effective turning points in the biological control of this invader. In a given habitat, the massive concentration of morphotype I specimens can, in fact, indicate a good adaptability to the environmental conditions, and a prosperous breeding period.

A full understanding of life history characteristics of invasive species is considered as a fundamental prerequisite for the development of management strategies [63], and in this perspective, our study could represent a user-friendly and low-cost tool to assess the reproductive status of *P. clarkii*, especially in discrete and isolated geographic areas. In such a context, the island of Sardinia, which represents a closed, geographically isolated system with typical Mediterranean features [64,65], characterized by a unique combination of geophysical, climatic, and biotic factors, as well as an exceptional susceptibility to invasions by species throughout history [66], has registered a massive occurrence of this crayfish [30] since 2005 [25]. Consequently, this Mediterranean island has become an ideal geographical model for studies devoted to shedding further light on the morphological variations typical of the reproductive phenotypes of *P. clarkii.* Therefore, the aim of this research is to supply the first statistical support to the description of the two morphotypes in *P. clarkii* captured in the island of Sardinia from a morphometric point of view, as additional data, to implement local management plans for this extremely invasive alien. To ensure a correct taxonomic attribution of the specimens collected for the morphological analysis, a molecular identification of the species has been performed in advance, based on mitochondrial markers. The primary objective of the molecular investigation was to prevent the species misidentification. Indeed, among the quiescent specimens classified as form II, it is not possible to recognize the taxonomic reference points that describe the holotype in the species *P. clarkii*, which are, on the contrary, well visible in the specimens classified as form I. This fact could result in incorrect species recognition, in particular among juvenile female specimens of the American crayfish (form II) and its congener marbled crayfish, *Procambarus virginalis*, Lyko, 2017. The latter species was, in fact, recently reported in Sardinia [67]. Furthermore, a molecular diagnostic check to evidence the presence or absence of the pathogen, *Aphanomyces astaci,* was also carried out based on a nuclear ribosomal molecular marker.

## 2. Materials and Methods

### 2.1. Sampling and Biological Data Collection

**Figure 1:**
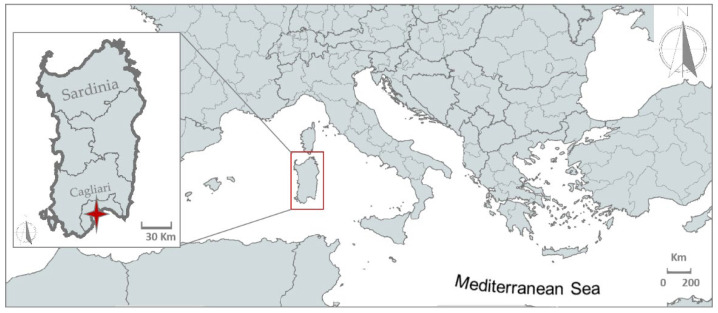
The red star on Sardinia Island in the map indicates the sampling site in the Metropolitan District of Cagliari were Procambarus clarkii individuals were collected.

From July to October 2023, a total of 75 specimens were caught in the southern region of Sardinia corresponding to the Metropolitan area of Cagliari (Figure 1). The capture process was conducted in the Flumini Mannu river, characterized by lotic waters; it is the main natural watercourse tributary to the Santa Gilla Pond. It starts in contiguous artificial and natural waterways created for the flow of water. The sampling was performed by placing double-entry cylindrical crayfish traps (50 cm in length and 20 cm in diameter, with a 20 mm mesh, Trappy, 10 st Art nr 1010, made in Sweeden, https://www.trappy.com/en/index-page/, accessed on 2 May 2023) in wet riverbed areas. Each trap included a bait box (baited with wet food for dogs and cats) inside and was attached to a line to facilitate easy retrieval. Every trap was labeled with a unique identification number on a rigid plastic tag. The traps were left active for a period of five days, with daily monitoring and bait replacement following the initial setup day. Further information about the sampling are provided in Supplementary Table S1, along with a detailed map of the sampling areas and stations in Figure S1.

All the captured animals were sacrificed for further analysis by freezing them in order to lower the metabolic rates and minimize their suffering. The samples were preventively cooled to 4 °C and, successively, frozen at −18 °C. The above reported sampling method was approved by the Ethics Committee of the University of Sassari (Prot. n. 79992 of 16 July 2024).

**Figure 2:**
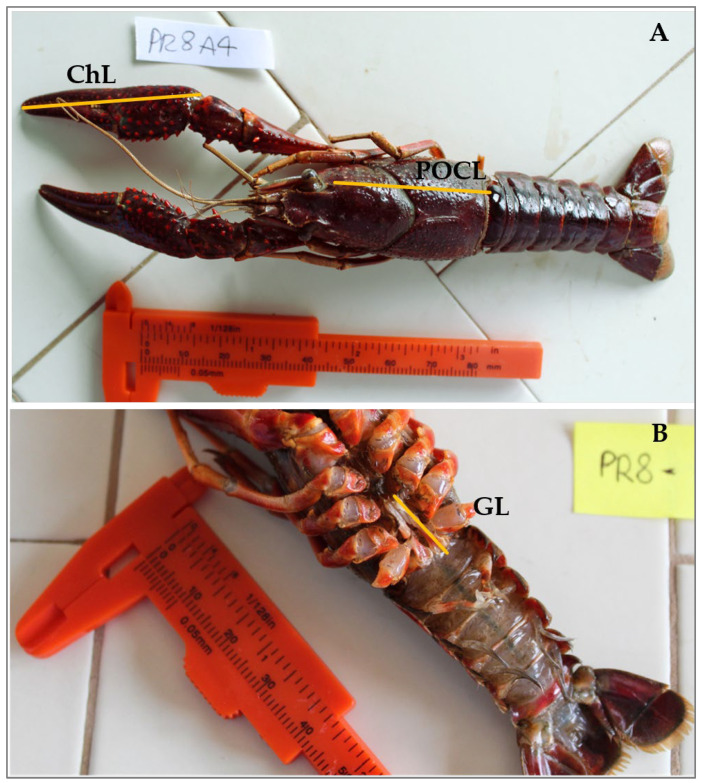
The picture illustrates the principal biometric measures in Procambarus clarkii. ChL, chela length; POCL, post-orbital carapace length, on dorsal side (**A**). Picture of the GL (gonopod length) on the ventral side (**B**).

For each specimen, the following measures were taken: (1) postorbital carapace length (POCL), (2) chelae length (ChL), (3) and gonopod length (GL) in males (Figure 2A,B). We used POCL to measure the body size of *P. clarkii*.

### 2.2. Molecular Analysis

#### 2.2.1. Taxonomic Identification of *Procambarus* spp.

Total genomic DNA was isolated from a portion of the soft abdominal tissue of the crayfish using the Macherey-Nagel Nucleo Spin Tissue Kit (MACHEREY-NAGEL GmbH & Co. KG, Düren, Germany), following the supplier’s instructions. DNA solutions were quantified using the Nanodrop™ Lite Spectrophotometer (by Thermo Scientific; Waltham, MA, USA), which showed an average yield of 24.4 ng/µL. PCRs were, then, performed using the following primers, specific for mitochondrial Cytochrome C subunit I gene [68]: COI Folmer FOR (forward) (5′-GGTCAACAAAATCATAAAGATATTGG-3′) and COI Folmer REV (reverse) (5′-TAAACTTCAGGGTGACCAAAAAATCA-3′). Reactions were carried out in a total volume of 25 µL. On average, 10 ng of total genomic DNA were combined with 0.6 µM of each primer and one pellet of PuReTaq Ready-To-Go PCR beads (GE Healthcare, Wauwatosa, WI, USA) containing stabilizers, 4 ng of bovine serum albumin (BSA), deoxynucleotide triphosphates (dNTPs), 2.5 units of PuReTaq DNA polymerase, and reaction buffer. When a bead was reconstituted to a 25 µL final volume, the concentration of each dNTP and MgCl_2_ was set at 200 µM and 1.5 mM, respectively. PCRs were performed in a GeneAmp PCR System 9700 Thermal Cycler (Applied Biosystems, Waltham, MA, USA), programmed as follows: 1 cycle of 4 min at 94 °C, 35 cycles of 30 s at 94 °C, 30 s at 48 °C (annealing for COI gene primers), and 30 s at 72 °C. At the end, a post-treatment of 10 min at 72 °C and a final cooling at 4 °C were carried out. Both positive (high-quality DNA samples from the species *Procambarus clarkii*) and negative controls were used to test the effectiveness of the PCR protocols and the absence of possible contaminations. Electrophoresis was carried out on 2% agarose gels, prepared using 1× TAE buffer (Tris-Acetate-EDTA, pH 8.3) and stained with Gel Red Nucleic Acid Stain (Biotium Inc., Fremont, CA, USA). The PCR products were purified by ExoSAP-IT (USB Corporation, Cleveland, OH, USA) and sequenced for forward and reverse strands (by means of the same primers used for PCR), using an external sequencing core service (Macrogen Europe, Milan, Italy). The total 75 sequences obtained were aligned using the package Clustal Omega [69] (available at https://www.ebi.ac.uk/jdispatcher/, accessed on 10 September 2024) and deposited on GenBank (accession numbers: PQ365549-PQ365552). With the use of software DNA Sequence Polymorphism Version 6.12.03 Copyright© 1995–2018, University of Barcelona [70], the total number of haplotypes within the sequences was analyzed.

#### 2.2.2. Diagnostic Analysis for the Pathogen, *Aphanomyces astaci*

For the diagnostic investigation of the presence of the pathogenic oomycete, *Aphanomyces astaci*, DNA extracted from both the animal tissue (abdominal portion) and swabs from the ventral side and darkened areas on the chelae and telson of each specimen were analyzed. The DNA was extracted following the MACHEREY-NAGEL method described above. DNA solutions were quantified using the Nanodrop™ Lite Spectrophotometer, which yielded an average of 10 ng/µL.

PCRs were performed using the following primers, specific to the Internal Transcribed Spacer region surrounding the 5.8 rRNA gene: ITS 1A (forward) (5′-TCCGTAGGTGAACCTGCGG-3′) and ITS 4A (reverse) (5′-TCCTCCGCTTATTGATATGC-3′) [71,72]. For each PCR reaction, the GeneAmp PCR System 9700 Thermal Cycler (Applied Biosystems, Waltham, MA, USA), was set as follows: 1 cycle of 4 min at 94 °C, 35 cycles of 30 s at 94 °C, 30 s at 51 °C (annealing for ribosomal ITS primers), and 30 s at 72 °C. The PCR products that showed a potentially indicative result of the presence of the pathogen during electrophoresis (the occurrence of a band which was approximately 750 base pairs long) were purified and then sent to an external service center for nucleotide sequencing (Macrogen Europe, Milan, Italy).

### 2.3. Population Structure

The size-frequency distribution of the entire sampled population, divided by sex, was calculated for each size class (1 mm POCL), and the normality of the distribution was tested using an F-test [73]. Differences in the size composition between sexes was investigated through the Student’s t-test.

The sex ratio (MM/FF + MM) was performed and differences in the expected ratio 1:1 between sexes was investigated using the Chi-squared test (ꭓ2).

### 2.4. Morphotypes Analysis

#### Intra- and Inter-Sexual Dimorphism

**Figure 3:**
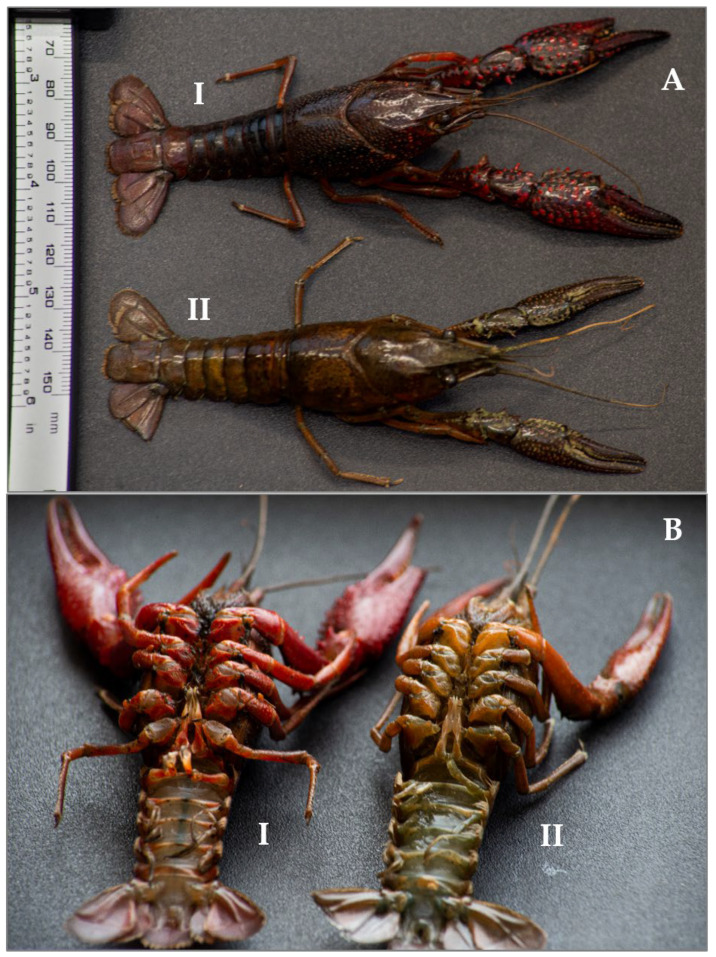
The picture illustrates the dorsal view of two morphotypes (I and II) of Procambarus clarkii specimens of the same size (33.6 mm POCL) (**A**). Ventral side of the two specimens (**B**). Pictures by Nicolò Giulianetti.

The samples were analyzed in order to establish sex and reproductive status. Males and females were identified on the basis of the presence/absence of the first two pairs of calcified pleopods, called gonopods, which are the secondary sexual characters in males [53,55,56,58,59] (Figure 3), and on the position of the gonopores, at the coxae of the third pereopods in females [56].

Males were, then, assigned to each form (I or II) on the basis of chelae length size, hooks, and pleopod hardness [58,59,60]. Female specimens were also divided on the basis of the chela length size and the presence of a cornified annulus ventralis in form I females [59]. Suko [53], and years later, Hamasaki et al. [58], in fact, stated that in *P. clarkii* females, the reproductive form I has longer chelae compared to those of same-sized specimens in form II. Furthermore, we could distinguish the morphotypes through the presence of a different color pattern: redder in form I, brownish or green in form II for both sexes [60].

The intra-sexual dimorphism within the morphotypes of the two separated sexes was evaluated with the use of allometric analysis. The relative growth of the body part of interest (ChL) with respect to a reference dimension (POCL) was examined using the following Equation (1) [74]:

y = ax^b^ (1)

where x is the main body size (POCL), y is the measurement for another body-part (ChL), b is the allometric growth coefficient, and a is the initial growth constant. The relative growth patterns could be defined as follows: b > 1 indicates positive allometric growth, where y grows faster than x; b = 1 indicates isometric growth, where y and x exhibit the same growth rate; and b < 1 indicates negative allometric growth, where y grows slower than x. The growth equation parameters were estimated through a linear model lny = lna + blnx (lm function) of the log-transformed data lengths (POCL and ChL). The right and left chelae were symmetrical (ANOVA *p*-value > 0.05), and some specimens appeared to have regenerating smaller right or left chelipeds. Therefore, we selected the larger one to take measurements, following [58]. The differences in the regression lines of the two morphotypes were tested for each sex using an F-test [75].

The normality of distribution in the collected chelae length data was tested through the Shapiro–Wilk test [76], and the homoscedasticity of variance was tested using the Bartlett’s test [77]. The mean values of ChL were analyzed using Student’s *t*-test to identify any significant differences between the average values of the male and female morphotypes [78], thereby assessing the presence of intra-sexual dimorphism.

After determining the existence of any differences between the means, a post hoc Tukey’s test was used to determine which group (I or II) was responsible for the significant results [79].

Finally, the Wilcoxon test was run to detect the presence of inter-sexual dimorphism.

All the statistical analyses were performed on RStudio 2024.04.2 Build 764 © 2009–2024 Posit Software, PBC.

### 2.5. SOM Estimation

The estimation of the size at onset of maturity was conducted exclusively in males, as we found it to be a promising method to infer maturity in male sex, for which the direct gonads observation is often unsatisfactory. In many crustacean species, the estimation of size at maturity is based on the analysis of female maturity ogives, a classical method that relies on the direct observation of gonadal development [80,81]. In male decapods, instead, maturity cannot be readily determined from the macroscopic examination of gonads and associated structures, and few such studies have been carried out (e.g., [82,83]). Therefore, in order to estimate the size at onset of maturity (SOM), we employed the same allometric function (1) [84], using the two variables POCL and GL of the reproductive (MI) and non-reproductive (MII) forms, and their values were logarithmized (ln y = ln a + bln x). In this case, x represents the independent variable (POCL), y is the dependent variable (GL), a is the intercept, and b the slope of the regression line. In the allometric growth, the carapace length (POCL) was used as the independent variable (x), since it is the most representative dimension of the overall size of the animal and is related to other body dimensions. The dependent variable (y) was gonopod length (GL).

The appearance of an inflection point of the regression lines should represent the size at which the crayfish undergoes the ecdysis process to reach maturity, and therefore, the reproductive morphotype I. The strucchange package [85] was used to identify the breakpoint in the relationship between POCL and GL. This approach detects structural changes in linear regression models.

The function breakpoints () was applied to the entire dataset to calculate the confidence intervals for the estimated breakpoint.

After identifying the breakpoint, the dataset was divided into two segments based on the breakpoint value. Separate linear regression models were fitted to each segment using lm(), and the slopes were compared using an ANCOVA test package (“car”, version 3.1-3) [86].

## 3. Results

### 3.1. Molecular Analysis

#### Taxonomic Identification of *Procambarus* spp. Individuals and Diagnostic Analysis of *Aphanomyces astaci*

A total of 75 sequences from the COI region (630 bp long) were obtained in the present study. All the newly generated sequences were identified as belonging to the species *Procambarus clarkii* through Basic Local Alignment Search Tool (BLAST) analysis, implemented in the GenBank nucleotide database (https://www.ncbi.nlm.nih.gov/genbank/ accessed on 2 October 2024), which showed the presence of four distinct haplotypes and a percentage of identity ranging from 99.84% to 100% for the COI (Table 1). All the further information regarding the collected samples is provided in Supplementary Table S2. Concerning the presence of *Aphanomyces astaci*, the samples that showed a potentially indicative presence of the pathogen during electrophoresis (approximately 750 base pair long), tested negative for the oomycete after the sequencing analysis.

### 3.2. Population Structure

**Figure 4:**
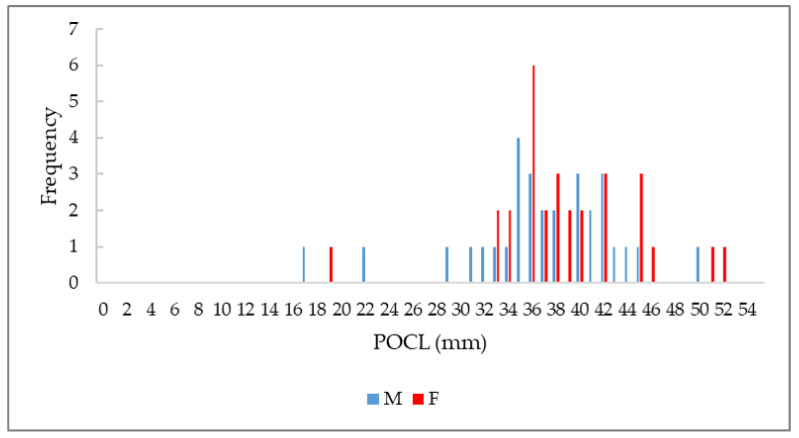
Graphic representation of Procambarus clarkii population size-structure (mm) in males and females.

The sampled population showed a Gaussian distribution, with the minimum and maximum values in males (n. 38) and females (n. 37) as follows: 49.6–16.8 mm POCL (average ± s.d = 36.4 ± 6.42) and 51.5–18.3 mm POCL (average ± s.d = 38.3 ± 6.19) (Figure 4).

The size-distribution did not differ significantly between sexes (Student’s *t*-test: *p*-value = 0.23; F-test: *p*-value = 0.84), with a sex ratio equal to 1.03, and a ꭓ2 test that was not statistically significant (ꭓ2 = 0.0169, d.f. = 1, *p*-value = 0.89).

### 3.3. Morphotypes’ Analysis: Inter- and Intra-Sexual Dimorphism

**Figure 5:**
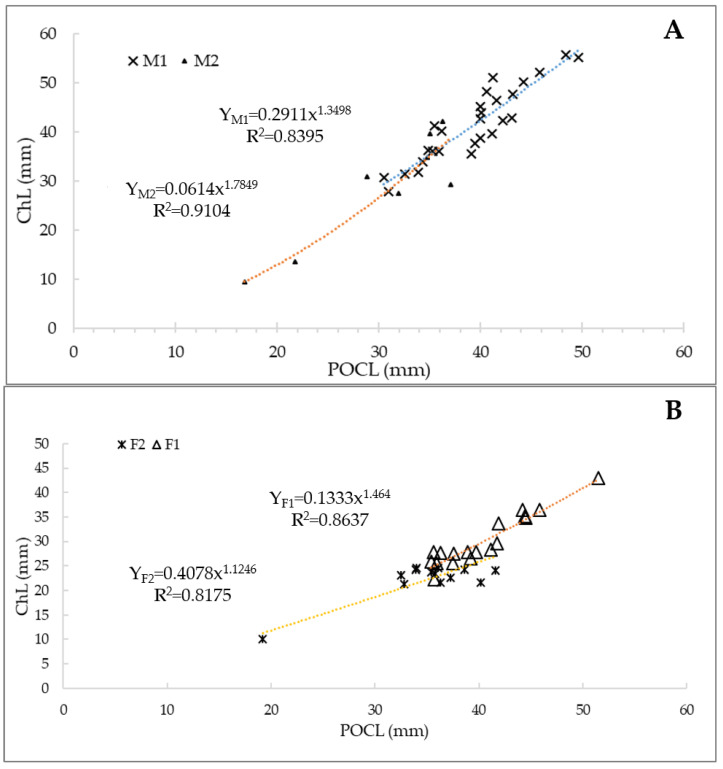
Graphic representation of the power function with the two variables (POCL and ChL) in Procambarus clarkii males (**A**) and females (**B**). The different colors of the trendlines are useful to distinguish the morphotypes I (blue for males and orange for females) and II (orange for males and yellow for females).

The two different morphotypes were analyzed through the allometric growth parameters, detecting the relationship between POCL and another dependent variable (ChL) (Figure 5A,B).

Both sexes’ power equations were significant (linear model MI: lny = −1.2345 + 1.3498⋅lnx, R^2^ = 0.92; linear model MII: lny = −2.7912 + 1.7849⋅lnx and R^2^ = 0.96; linear model FI: lny = −2.0143 + 1.464⋅lnx and R^2^ = 0.86; linear model FII: lny = −0.8967 + 1.1246⋅lnx and R^2^ = 0.97; *p*-value < 0.05 in all cases), with an allometric growth coefficient of b > 1 in all the analyzed cases. The F-test showed significant differences between the regression lines of the two morphotypes in males (F-statistic: 24.157; F-value: 3.938; *p*-value: 3.57 × 10^−6^) and females (F-statistic: 19.054, F-value: 3.938, *p*-value: 3.15 × 10^−5^).

**Figure 6:**
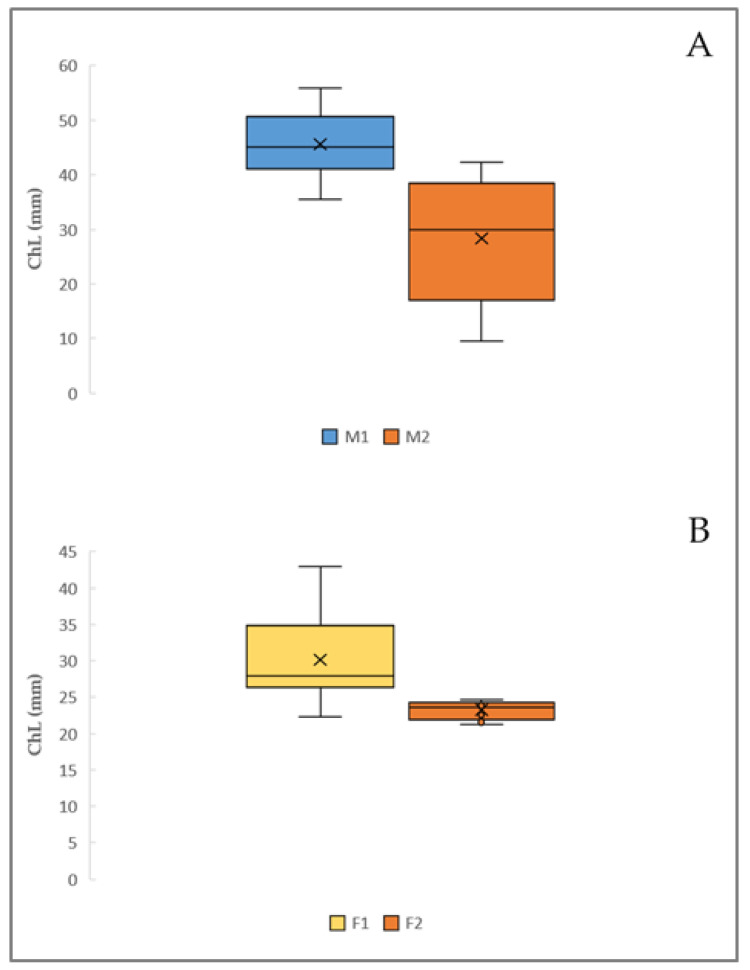
Graphic representation of the box plot with chela dimensions in the two morphotypes for males (**A**) and females (**B**). (**A**) Form I: mean = 45.6 mm; std = 6.009; min = 35.5 mm; Q1 = 42.3 mm; Q2 = 45.1 mm; Q3 = 50.2 mm; max = 55.8 mm; range = 20.29 mm; IQR = 7.90 mm. Form II: mean = 28.38 mm; std = 11.617 mm; min = 9.5 mm; Q1 = 23.92 mm; Q2 = 30.00; Q3 = 36.07; max = 42.2 mm; range = 32.7 mm; IQR = 12.15 mm. (**B**) Form I: mean = 27.84 mm; std = 2.205 mm; min = 25.6 mm; Q1 = 26.35 mm; Q2 = 27.75 mm; Q3 = 28.02 mm; max = 33.7 mm; range = 8.10 mm; IQR = 1.67 mm. Form II: mean = 23.26 mm; std = 1.198 mm; min = 21.3 mm; Q1 = 22.37 mm; Q2 = 23.6 mm; Q3 = 24.22; max = 24.6 mm; range = 3.30 mm; IQR = 1.85 mm.

The Shapiro–Wilk test showed a normal distribution in male chelae length samples (*p*-value = 0.12), but not for females (*p*-value = 0.001). For this reason, males’ samples were analyzed with the Student’s t-test for means values between the morphotypes, with the following results: *p*-value = 0.014. The homogeneity of variance was assessed using the Bartlett’s test, which showed that the variance between the two chelae length morphogroups was not statistically significant (*p*-value = 0.14). Finally, the post-hoc Tukey test’s *p*-value = 0.0006 highlighted that the difference in means values was due to higher values in MI ChL (Figure 6A).

**Figure 7:**
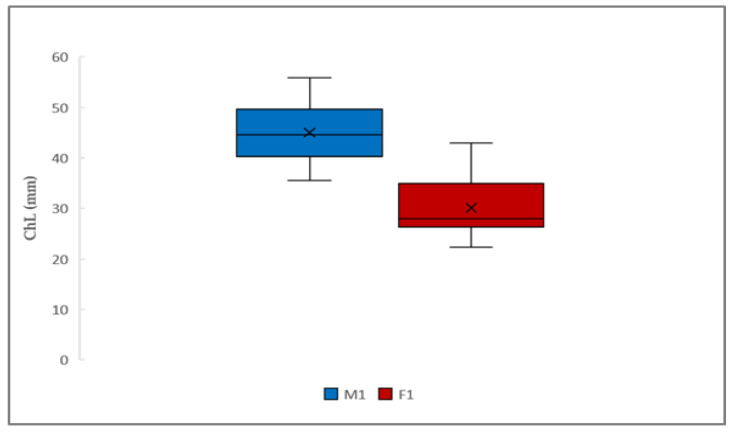
Graphic representation of the box plot with chela dimensions (ChL) in males (M1) and females (F1). Males: mean = 45.0 mm; std = 5.656; min = 35.5 mm; Q1 = 41.65 mm; Q2 = 44.55 mm; Q3 = 48.7 mm; max = 55.8 mm; range = 20.29 mm; IQR = 7.05 mm. Females: mean = 30.58 mm; std = 5.030 mm; min = 25.6 mm; Q1 = 27.5 mm; Q2 = 27.9 mm; Q3 = 34.8 mm; max = 43.0 mm; range = 17.4 mm; IQR = 7.29 mm.

For females, due to the non-normality of the data distribution, a non-parametric test, Wilcoxon–Mann–Whitney was performed in order to detect any differences between the medians of chelae length for the two morphotypes, with the following results: a highly significant *p*-value < 0.05. The higher median value was registered for the group FI (27.75 mm), while that for FII was equal to 23.6 mm (Figure 6B). Finally, in order to detect the inter-sexual dimorphism in the chela length of males and females, a Wilcoxon test was conducted, showing a *p*-value < 0.05 and higher values for males (Figure 7).

### 3.4. SOM Estimation

**Figure 8:**
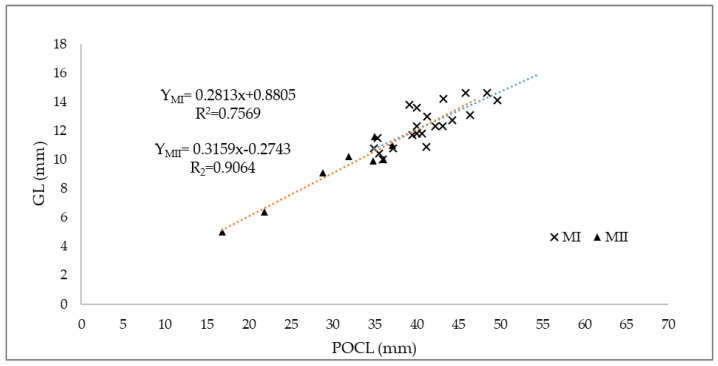
Graphic representation of the regression line between the two variables, carapace length, (POCL), and gonopod length (GL) in P. clarkii males. The different colors of the trendlines are useful to distinguish the morphotype I (blue) and II (orange).

The allometric growth relationship between the explicative variable POCL and the length of the secondary sexual character (gonopod length, GL), was used to identify the SOM (size at morphometric maturity), at which the animal could split from the non-reproductive form II to the reproductive morphotype I and reach maturity. In particular, both forms showed a strong correlation between the two variables (R^2^ = 0.75 and 0.90, in form I and II, respectively, with a *p*-value < 0.05) (Figure 8). A structural breakpoint in the relationship between POCL and GL was identified using the strucchange package. The breakpoints() function detected a significant change point at 35.0 mm POCL (95% confidence interval: 35.0, 37.1). Visual inspection of the segmented regression plot confirmed the transition between the two groups. The slopes before and after the breakpoint differed significantly (Δ slope = 0.035, *p* < 0.001), suggesting distinct gonopods growth patterns in morphotypes I and II.

## 4. Discussion

Invasive species are among the most relevant causes of extinctions [87], particularly in aquatic ecosystems [88]. Their intentional or inadvertent introductions have become more frequent than ever in the last decades, likely due to the effects of globalization, in particular, due to the increase in international trade [14,89,90]. In this work, we focused on some morphological aspects of a highly invasive crustacean, *Procambarus clarkii*, with the aim to characterize the two distinct morphotypes in the isolated Sardinian Mediterranean population, providing statistic support to morphometric analysis. An organism’s shape is defined by the sizes of its body parts (traits) in relation to the size of the whole (body size), with the scaling pattern relating trait size to body size known as the trait allometry [91]. Considering this fact, we put in relationship distinct parts of the crayfish bodies, in order to establish the existence of a connection between them, and find evidence of faster growth, relative to body size, of the dependent variables: chela (ChL) and gonopod (GL) lengths. Indeed, the technique of allometry provided new results for the SOM estimation of the crayfish, especially in males. Size at the onset of sexual maturity is, in fact, one of the most important parameters of the life cycle of crustaceans [92], and knowledge on the reproductive biology and population dynamics of invasive species is essential for environmental conservation and protection of native species [93].

This approach could be useful to identify the reproductive cycle of the invasive crayfish for which the two alternative morphotypes have been descripted within the reproductive cycle [53,54,55,56,57,58,59,94,95].

Molecular analysis conducted in the present study using the mitochondrial COI gene confirmed the taxonomic identity of the captured specimens as *P. clarkii*. The diagnostic method used to detect a possible infection by *Aphanomyces astaci* [71,72] did not highlight the presence of this harmful pathogen in the crayfish captured in the present study; in fact, all the analyzed specimens tested negative for the oomycete. This result could suggest the possible lack of the pathogen in the south of Sardinia, but further analyses would be required to delve deeper into this topic. The study of the size composition and the population sex ratio highlighted a typical Gaussian distribution and a general homogeneity between the POCL length-frequency distribution in males and females. This trend was perfectly in line with other previous studies conducted in the wetlands of central Italy (Latium and Tuscany), in which no differences in males’ and females’ sizes were detected [31,96]. However, other analysis on the size-structure performed in the Italian populations of the central Italy, Lake Trasimeno (Umbria) [22,97] and north-western Italy, Lake Candia (Piedmont) [98] revealed, instead, a significant difference in the carapace mean values (measure taken from the tip of the rostrum to the end of the carapace), with males larger than females and vice versa [22].

The complete lack of smaller size-classes ranging from 0 to 16 mm POCL may be attributed to the chosen capture method (trap bait). Smaller crayfish could potentially not be drawn to the bait due to their distinct dietary preferences compared to adults. Additionally, their limited mobility might result in less frequent abandonment of their burrows. Finally, it is plausible that, thanks to their reduced size, the small crayfishes could easily pass through the mesh of the trap [32,99].

We found that sex ratio did not significantly differ from the expected 1:1, as also observed by other authors (i.e., [31,32,97,100]). This result is in line with the current knowledge about wild populations of astacid crayfish in general, with sex ratios close to 1:1, consolidated polygamy [101,102] and consequently increased broodstock [102]. In the original North American area of distribution (Louisiana), the ratio of females to males calculated throughout the year was approximately equal, even though there was a considerable decrease in females during the warmer months, due to their annual spawning cycle [103,104].

On the contrary, instances of unbalanced sex ratios during different periods of the year have been reported in central (Latium; Umbria) and south—central (Sicily) Italian and insular populations of *P. clarkii*, with either males outnumbering females [21,97,105,106] or vice versa [96,105]. In Donato et al. [98], it is hypothesized that the discrepancy in sex ratio could be linked to the reproductive activity of females during the summer months, providing parental care to the offspring in burrows.

The allometric analysis of the captured specimens, along with the subsequent statistical analysis, showed evidence of the existence of two distinct morphotypes (intra-sexual dimorphism) within the samples captured in Sardinia and highlighted the inter-sexual dimorphism between the two genders for the chela length characteristic. Larger chelipeds confer several benefits to crayfish: specimens with large chelae are, in fact, less susceptible to predation [94] and can dominate similar-sized crayfish provided with smaller ones [94,107,108,109,110]. Furthermore, this physical feature would help establish dominance and access to females [94,108,110,111]. Large chelipeds might provide an advantage for form I males competing to acquire receptive females, being advantageous for maternal form I females defending eggs or juveniles in shelters [18,58,112].

The allometric relationship also represents a powerful tool used to estimate the size at onset of maturity (SOM) [84,113]. In crustaceans, the allometric relationships between body size and other body parts are used to estimate maturity, assuming that the secondary sexual characteristics appear and grow at different rates at mature and immature stages [84,114]. In our study, the relationship between the explicative variable POCL and the length of the secondary sexual character (gonopod length, GL), was realized in order to identify the SOM at which the animal could split from the non-reproductive form II to the reproductive one (form I). In particular, both the morphotypes showed a strong correlation between the two variables. The males in form II exhibited a size distribution of about 21 mm POCL (16.8–37.1 mm), while the males in form I where distributed within a 14 mm POCL size class range (34.9–48.4 mm). It is possible to notice a break in the regression lines between 35 and 37.1 mm POCL, values at which morphotype II starts to disappear and morphotype I becomes predominant.

In the literature, there is not a comparable method for the estimation of SOM applied to *P. clarkii*, and our result completely differs from those obtained in previous studies investigating maturity. For example, in Suko [53] and Hamasaki et al. [58], the size at onset maturity was about 21 mm POCL in both sexes and later, in the recent review by Hamasaki et al. [59], the minimum size of *P. clarkii* at sexual maturity was reported to occur at a POCL equal to 18–29 mm for males, based on the occurrence of the reproductive morphotype, and at a POCL of 25–34 mm for females, based on gonad maturation and physical features [22,58,59,100,103,115,116,117,118,119]. In other previous studies, such as Correia et al. [120], performed on individuals from Portugal, the estimated SOM was more similar to that of our results: 36.4 mm CL for males and 36.2 mm CL for females. The size at sexual maturity probably varies according to the environmental conditions in each locality: for instance, sexually active males are considerably smaller in stressed environments with erratic water level fluctuations, high population densities, poor water quality, and limited food supply compared to more favorable environments [59,120,121]. Moreover, it is important to underline that, the morphotype II group includes both adult males that have already reached maturity and are now quiescent, and juvenile males that are still immature [58]. The larger size class of adult quiescent males could, for this reason, shift the estimation of SOM to higher POCL values. On the other hand, high values of SOM in the crayfish could reflect a good level of adaptation to the Sardinian habitat by this invasive species. The island of Sardinia offers, in fact, a typical Mediterranean climate, with mild and rainy winters and warm and dry summers [64,65], providing perfect environmental conditions for the establishment of alien species, reflected in a shift of the SOM to higher values.

## 5. Conclusions

The present work aimed at providing new insights into the reproductive biology of *Procambarus clarkii* by approaching it from a morphometric perspective. Furthermore, since the complex morphology of these crayfish and the possibility of multiple development stages often makes species identification misleading, molecular techniques were coupled to morphometric analysis, in order to validate the recognition achieved on a morphological basis. In addition, molecular diagnostic tools were also useful for revealing the complete absence of the highly pathogenic oomycete, *Aphanomyces astaci*, within our samples. Even though our study represents a preliminary investigation, the results obtained allow us (1) to provide statistical support to the presence of two distinct morphotypes in the Louisiana crayfish inhabiting the Mediterranean island of Sardinia and (2) to estimate the primary data on maturity for males of this species. Based on the first contribution of this study, two new research studies are being performed by these authors. The first aims to establish a significant connection between the two morphotypes and the different color patterns detected in the Louisiana crayfish, to deepen biological knowledge of this highly invasive crustacean. The second aims to corroborate the pattern evidenced by the present study, analyzing a larger number of specimens to provide further statistical significance to the occurrence of the two different morphotypes in western Mediterranean. Remarkably, the contemporary presence of these two morphotypes of *P clarkii* on Sardinia Island could also explain the misidentification that often occurs with the congeneric species *P. virginalis*, a “new entry” among the alien species of Sardinia [67], which shares its habitat with the Louisiana crayfish. *Procambarus clarkii*, during its non-reproductive phase, exhibits, in fact, distinct color patterns and markings which, especially in cases of sympatry, can lead to taxonomic misidentification with *P. virginalis*. During the external anatomy observation of the captured specimens, it was not unusual to find young individuals with peculiar, marbled shades on their lateral side and telson, and the poorly developed carapace made it difficult to identify the distinctive character of the areola in a hypothetical *P. virginalis* (n.p. pers. obs.). These two morphotypes have been previously described in other Italian regions, and it would be of interest in forthcoming studies to investigate whether the typical biotic and abiotic conditions of this Mediterranean island may serve as a selective booster for these morphotypes, increasing the presence and frequency of one over the other. Furthermore, a differential analysis between European and non-European morphotypes in the future could better clarify the role that environmental selection may have played on this species in the Mediterranean.

## Figures and Tables

**Table 1:** Total haplotypes detected among the 75 *P. clarkii* samples and Haplotype diversity (h), followed by the GenBank Accession number (GenBank code), Sampling site, Percentage of Identity with the species *Procambarus clarkii* (% identity), the Absolute and Relative haplotypes frequencies.

Haplotype	GenBank Code	Sampling Site	% Identity	Absolute Frequence	Relative Frequence
A	PQ365549	South Sardinia	100%	29	38.66%
B	PQ365550		99.84%	9	12%
C	PQ365551		99.84%	31	41.33%
D	PQ365552		100%	6	8%
h	0.58		Total	75	100%

## Data Availability

The sequences obtained in the present study for the mitochondrial Cy-tochrome c Oxidase subunit I gene isolated in Sardinian crayfish were deposited in the GenBank database under the accession numbers PQ365549-PQ365552. All the specimens analyzed in the present work are stored at the Conservation Genetics laboratory, Department of Veterinary Medicine of the University of Sassari, Italy.

## Supplementary Materials

The following supporting information can be downloaded at: https://www.mdpi.com/article/10.3390/ani14243558/s1. Table S1: GPS coordinates of the 15 sampling stations. Figure S1: Intervention areas for the capture of P. clarkii; the survey was developed in eight sites, within which a total of 15 stations were defined. Details of the 15 station geographic coordinates are given in Table S1. Table S2: Additional information about Procambarus clarkii specimens involved in genetic analyses.
