# Supplementary material for: New Insight into the Crayfish Procambarus clarkii (Girard, 1852) (Crustacea, Cambaridae): A Morphometric Combined Approach to Describe the Case of a Mediterranean Population
Source: Animals (Basel). 2024 Dec 10;14(24):3558. doi: 10.3390/ani14243558 (PMC11672703; doi:10.3390/ani14243558)

Figure S1. Intervention areas for the capture of *P. clarkii*; the survey was developed in 8 sites, within which a total of 15 stations were defined. Details for the geographic coordinates of the 15 stations are given in Table S1.

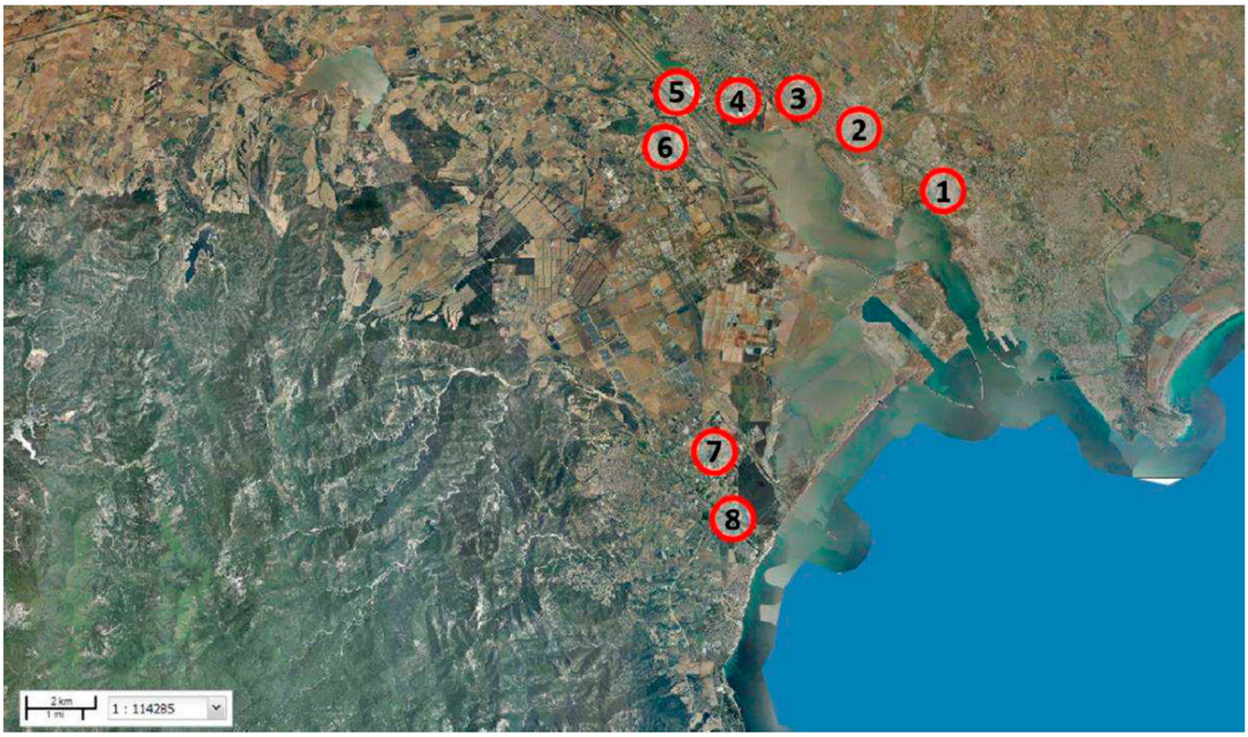

Supplement: Supplementary file 1 [file animals-14-03558-s001.zip › animals-3310944-supplementary/Figure S1.pdf]
