# Supplementary material for: New Insight into the Crayfish Procambarus clarkii (Girard, 1852) (Crustacea, Cambaridae): A Morphometric Combined Approach to Describe the Case of a Mediterranean Population
Source: Animals (Basel). 2024 Dec 10;14(24):3558. doi: 10.3390/ani14243558 (PMC11672703; doi:10.3390/ani14243558)
Supplement: Supplementary file 1 [file animals-14-03558-s001.zip › animals-3310944-supplementary/Table S1.pdf]

Table S1. GPS coordinates of the 15 sampling stations.

Station code	GPS coordinates	
	Latitude	Longitude
SGL_01	39°15'0.80"N	9° 4'55.74"E
SGL_02	39°15'45.28"N	9° 2'59.47"E
SGL_03	39°16'13.26"N	9° 2'5.10"E
SGL_04	39°16'58.37"N	9° 1'50.09"E
SGL_05	39°16'28.52"N	9° 0'30.07"E
SGL_06	39°16'39.48"N	8°59'41.98"E
SGL_07	39°16'28.73"N	8°59'47.45"E
SGL_08	39°16'29.57"N	8°59'36.36"E
SGL_09	39°16'30.46"N	8°59'30.38"E
SGL_10	39°16'16.80"N	8°59'34.86"E
SGL_11	39°16'17.86"N	8°59'7.47"E
SGL_12	39°15'40.79"N	8°59'29.02"E
SGL_13	39°10'29.12"N	9° 0'41.03"E
SGL_14	39° 9'55.16"N	9° 0'24.86"E
SGL_15	39° 9'33.99"N	9° 0'52.32"E
