# Supplementary material for: New Insight into the Crayfish Procambarus clarkii (Girard, 1852) (Crustacea, Cambaridae): A Morphometric Combined Approach to Describe the Case of a Mediterranean Population
Source: Animals (Basel). 2024 Dec 10;14(24):3558. doi: 10.3390/ani14243558 (PMC11672703; doi:10.3390/ani14243558)
Supplement: Supplementary file 1 [file animals-14-03558-s001.zip › animals-3310944-supplementary/Table S2.pdf]

Table S2: Additional information about *Procambarus clarkii* specimens involved in genetic analyses.

Station	Sample code	Sampling site	Sampling date	Species	*Aphanomyces astaci*	Sex	Haplotype	Morphotype
2	PR2A1	South Sardinia	10/07/2023	*P. clarkii*	negative	M	PQ365549	1
6	PR6 A1	South Sardinia	08/07/2023	*P. clarkii*	negative	F	PQ365549	1
6	PR6 B1	South Sardinia	10/07/2023	*P. clarkii*	negative	M	PQ365549	1
7	PR7 A1	South Sardinia	13/10/2023	*P. clarkii*	negative	F	PQ365549	1
7	PR7 D3	South Sardinia	14/10/2023	*P. clarkii*	negative	M	PQ365549	1
7	PR7 F1	South Sardinia	09/07/2023	*P. clarkii*	negative	M	PQ365549	1
7	PR7 F2	South Sardinia	09/07/2023	*P. clarkii*	negative	M	PQ365549	1
8	PR8 A2	South Sardinia	09/07/2023	*P. clarkii*	negative	M	PQ365549	1
8	PR8 A4	South Sardinia	09/07/2023	*P. clarkii*	negative	M	PQ365549	1
8	PR8 A6	South Sardinia	09/07/2023	*P. clarkii*	negative	F	PQ365549	1
8	PR8 A7	South Sardinia	09/07/2023	*P. clarkii*	negative	M	PQ365549	1
8	PR8 B2	South Sardinia	10/07/2023	*P. clarkii*	negative	M	PQ365549	1
8	PR8 B4	South Sardinia	10/07/2023	*P. clarkii*	negative	F	PQ365549	1
8	PR8 B5	South Sardinia	10/07/2023	*P. clarkii*	negative	F	PQ365549	2
8	PR8 C1	South Sardinia	11/07/2023	*P. clarkii*	negative	M	PQ365549	1
8	PR8 C2	South Sardinia	11/07/2023	*P. clarkii*	negative	F	PQ365549	1
8	PR8 D1	South Sardinia	12/10/2023	*P. clarkii*	negative	M	PQ365549	1
8	PR8 E1	South Sardinia	13/10/2023	*P. clarkii*	negative	M	PQ365549	1
13	PR13 A2	South Sardinia	09/07/2023	*P. clarkii*	negative	F	PQ365549	2
13	PR13 A3	South Sardinia	09/07/2023	*P. clarkii*	negative	M	PQ365549	2
13	PR13 A4	South Sardinia	09/07/2023	*P. clarkii*	negative	F	PQ365549	1
13	PR13 A5	South Sardinia	09/07/2023	*P. clarkii*	negative	M	PQ365549	1
13	PR13 A6	South Sardinia	09/07/2023	*P. clarkii*	negative	M	PQ365549	1
13	PR13 A7	South Sardinia	09/07/2023	*P. clarkii*	negative	F	PQ365549	2
13	PR13 A8	South Sardinia	09/07/2023	*P. clarkii*	negative	F	PQ365549	1
13	PR13 B1	South Sardinia	10/07/2023	*P. clarkii*	negative	F	PQ365549	1

13	PR13 C1	South Sardinia	11/07/2023	*P. clarkii*	negative	F	PQ365549	1
13	PR13 C2	South Sardinia	11/07/2023	*P. clarkii*	negative	M	PQ365549	1
13	PR13 C3	South Sardinia	11/07/2023	*P. clarkii*	negative	F	PQ365549	1
7	PR7F1	South Sardinia	09/07/2023	*P. clarkii*	negative	M	PQ365550	1
7	PR7F2	South Sardinia	09/07/2023	*P. clarkii*	negative	M	PQ365550	1
8	PR8 A1	South Sardinia	09/07/2023	*P. clarkii*	negative	F	PQ365550	1
8	PR8 A3	South Sardinia	09/07/2023	*P. clarkii*	negative	F	PQ365550	1
8	PR8 A5	South Sardinia	09/07/2023	*P. clarkii*	negative	F	PQ365550	2
8	PR8F1	South Sardinia	08/07/2023	*P. clarkii*	negative	M	PQ365550	1
8	PR8F2	South Sardinia	08/07/2023	*P. clarkii*	negative	M	PQ365550	1
8	PR8F3	South Sardinia	08/07/2023	*P. clarkii*	negative	M	PQ365550	1
11	PR11 A2	South Sardinia	12/10/2023	*P. clarkii*	negative	M	PQ365550	1
6	PR6 C1	South Sardinia	12/10/2023	*P. clarkii*	negative	F	PQ365551	2
6	PR6 D1	South Sardinia	13/10/2023	*P. clarkii*	negative	F	PQ365551	2
6	PR6 E1	South Sardinia	15/10/2023	*P. clarkii*	negative	F	PQ365551	2
7	PR7 B1	South Sardinia	12/10/2023	*P. clarkii*	negative	F	PQ365551	1
7	PR7 B2	South Sardinia	12/10/2023	*P. clarkii*	negative	M	PQ365551	2
7	PR7 C1	South Sardinia	13/10/2023	*P. clarkii*	negative	M	PQ365551	2
7	PR7 C3	South Sardinia	13/10/2023	*P. clarkii*	negative	F	PQ365551	2
7	PR7 D1	South Sardinia	14/10/2023	*P. clarkii*	negative	M	PQ365551	1
7	PR7 D2	South Sardinia	14/10/2023	*P. clarkii*	negative	M	PQ365551	1
7	PR7 E1	South Sardinia	15/10/2023	*P. clarkii*	negative	M	PQ365551	2
7	PR7 E2	South Sardinia	15/10/2023	*P. clarkii*	negative	F	PQ365551	1
7	PR7 E3	South Sardinia	15/10/2023	*P. clarkii*	negative	M	PQ365551	2
7	PR7 E4	South Sardinia	15/10/2023	*P. clarkii*	negative	M	PQ365551	2
8	PR8 B1	South Sardinia	10/07/2023	*P. clarkii*	negative	F	PQ365551	1
8	PR8 B3	South Sardinia	10/07/2023	*P. clarkii*	negative	F	PQ365551	1
8	PR8F4	South Sardinia	08/07/2023	*P. clarkii*	negative	F	PQ365551	1

8	PR8F5	South Sardinia	08/07/2023	*P. clarkii*	negative	M	PQ365551	1
8	PR8F6	South Sardinia	08/07/2023	*P. clarkii*	negative	M	PQ365551	1
8	PR8F7	South Sardinia	08/07/2023	*P. clarkii*	negative	M	PQ365551	1
8	PR8F8	South Sardinia	08/07/2023	*P. clarkii*	negative	M	PQ365551	1
10	PR10 A1	South Sardinia	13/10/2023	*P. clarkii*	negative	M	PQ365551	1
10	PR10 A2	South Sardinia	13/10/2023	*P. clarkii*	negative	F	PQ365551	2
10	PR10 A3	South Sardinia	13/10/2023	*P. clarkii*	negative	F	PQ365551	2
10	PR10 B1	South Sardinia	14/10/2023	*P. clarkii*	negative	F	PQ365551	1
10	PR10 B2	South Sardinia	14/10/2023	*P. clarkii*	negative	F	PQ365551	1
10	PR10 B3	South Sardinia	14/10/2023	*P. clarkii*	negative	M	PQ365551	1
11	PR11 A1	South Sardinia	12/10/2023	*P. clarkii*	negative	M	PQ365551	1
11	PR11 B1	South Sardinia	13/10/2023	*P. clarkii*	negative	M	PQ365551	2
11	PR11 C1	South Sardinia	15/10/2023	*P. clarkii*	negative	F	PQ365551	1
11	PR11 C2	South Sardinia	15/10/2023	*P. clarkii*	negative	M	PQ365551	1
12	PR12 A1	South Sardinia	11/07/2023	*P. clarkii*	negative	F	PQ365551	2
7	PR7 C2	South Sardinia	13/10/2023	*P. clarkii*	negative	F	PQ365552	2
10	PR10 C1	South Sardinia	10/07/2023	*P. clarkii*	negative	F	PQ365552	2
10	PR10 C2	South Sardinia	10/07/2023	*P. clarkii*	negative	F	PQ365552	2
13	PR13 A1	South Sardinia	09/07/2023	*P. clarkii*	negative	F	PQ365552	1
13	PR13 C4	South Sardinia	11/07/2023	*P. clarkii*	negative	F	PQ365552	2
7	P07 fallax(?)	South Sardinia	14/10/2023	*P. clarkii*	negative	F	PQ365552	2
